# The Application of Organic Semiconductor Materials in Spintronics

**DOI:** 10.3389/fchem.2020.589207

**Published:** 2020-10-22

**Authors:** Yixiao Zhang, Lidan Guo, Xiangwei Zhu, Xiangnan Sun

**Affiliations:** ^1^Key Laboratory of Nanosystem and Hierarchical Fabrication, Chinese Academy of Sciences (CAS) Center for Excellence in Nanoscience, National Center for Nanoscience and Technology, Beijing, China; ^2^Center of Materials Science and Optoelectronics Engineering, University of Chinese Academy of Sciences, Beijing, China; ^3^School of Materials Science and Engineering, Zhengzhou University, Zhengzhou, China

**Keywords:** organic spintronics, π-conjugated semiconductor, spin transport, multifunctional spintronic device, spin manipulation, spin valve

## Abstract

π-Conjugated semiconductors, primarily composed of elements with low atomic number, are regarded as promising spin-transport materials due to the weak spin–orbit coupling interaction and hence long spin relaxation time. Moreover, a large number of additional functions of organic semiconductors (OSCs), such as the abundant photo-electric properties, flexibility, and tailorability, endow the organic spintronic devices more unique properties and functionalities. Particularly, the integration of the photo-electric functionality and excellent spin transport property of OSCs in a single spintronic device has even shown great potential for the realization of spin manipulation in OSCs. In this review, the application of OSCs in spintronic study will be succinctly discussed. As the most important and extensive application, the long-distance spin transport property of OSCs will be discussed first. Subsequently, several multifunctional spintronic devices based on OSCs will be summarized. After that, the organic-based magnets used for the electrodes of spintronic devices will be introduced. Finally, according to the latest progress, spin manipulation in OSCs via novel spintronic devices together with other prospects and challenges will be outlined.

## Introduction

A huge revolution has been made in the field of information storage since the discovery of the giant magnetoresistance effect and the development of spintronics in the past of 30 years (Baibich et al., 1988; Wolf et al., 2001). Organic semiconductors (OSCs) composed of light elements have weak spin–orbit coupling (SOC) interaction and thus long spin relaxation time with second level (Boehme and Lupton, 2013). According to the present researches, it has been shown that there is a great potential of excellent spin transport characteristic of OSCs at room temperature (Sun et al., 2013; Zhang et al., 2013; Guo et al., 2019b). In addition, the abundant functionalities of OSCs and interfacial properties between ferromagnetic electrodes and OSCs have further increased the application modes of OSCs in spintronics, which have attracted wide attention in the areas of chemistry, materials, and physics (Guo et al., 2019a).

In this review, the application strategies of OSCs in spintronic study will be summarized succinctly. First, spin transport as the most important application of OSCs in spintronics will be introduced. Second, combined with the unique properties of OSCs, several functional spintronic devices will be introduced, including spin memory devices, spin photoresponse devices, spin photovoltaic devices, spin organic light-emitting diodes (spin-OLED), and flexible spin devices. Third, organic-based magnets which can be used as magnetic electrodes will be introduced. Finally, the prospects and challenges for realizing spin manipulation in OSCs will be discussed.

## Application of OSCs in Spin Transport

**Figure 1:**
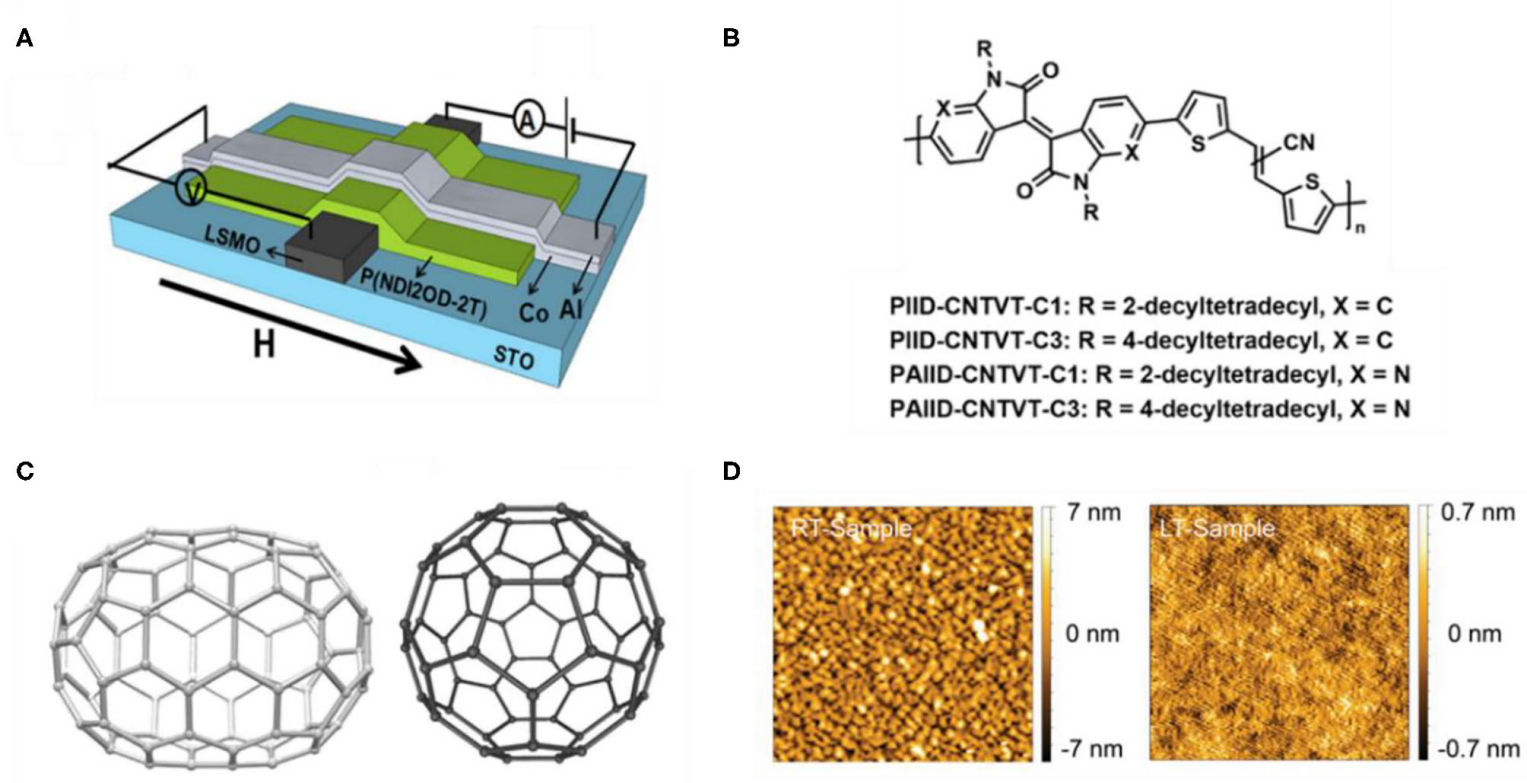
**(A)** Structure of a spin valve (Li et al., 2015). **(B)** Four conjugated polymers (Li et al., 2019). **(C)** C_70_ and C_60_ with different curvatures (Liang et al., 2016). **(D)** AFM images of 90-nm-thick F_16_CuPc films deposited under room temperature and low-temperature conditions (Sun et al., 2016b).

The long spin relaxation time of OSCs gives a great advantage in spin transport, and such advantage also constitutes the basis of functional spintronic devices. Spin valve is one of the most typical devices for spin transport study, which is composed of a spin-transport layer sandwiched between two ferromagnetic electrodes (Figure 1A) (Dediu et al., 2009; Sun et al., 2014a). The spin-polarized electrons are injected from one of the electrodes and transport in OSC thin film; finally, they are detected from another electrode (Jang and Richter, 2017). With sweeping the external magnetic field to configure the magnetization direction of the two electrodes as parallel (P) or antiparallel (AP), the device resistance can be tuned as low- (R_P_) or high-resistance states (R_AP_), respectively, which is the so-called spin valve effect and expressed by magnetoresistance [MR = (R_AP_ – R_P_)/R_AP_]. Such spin valve effect is the important evidence for demonstrating that the spin-polarized electrons have been successfully transported in OSC thin film, and the thickness of OSC can be considered as the spin transport length of OSCs (Sun et al., 2013; Zhang et al., 2013). It is worth noting that the aforementioned conclusion must be based on reliable spin valve preparation, that is, the soft OSC thin films should be ensured without the penetration resulting from the metal atoms during the device fabrication.

Based on the reliable spin valves, a series of studies on the molecular structure, composition, and aggregation structure of OSCs can be carried out, which have critical influence on the spin-transport performance. For instance, Li et al. have designed four conjugated polymers with analogous structures based on isoindigo (IID) units and researched the relation between molecular structure and spin transport property by measuring the spin valve effect (Figure 1B). They found that the introduction of pyridinic nitrogen can improve the MR value; however, the extent of alkyl chain branching points gets the opposite result (Li et al., 2019). Moreover, the effect of molecular curvature degree on spin relaxation time and spin transport length has been discussed. Liang et al. have observed that the spin transport length in the C_70_ thin film is apparently longer than that in C_60_ thin film at all temperatures through fabricating reliable carbon-based organic spin valves (Figure 1C) (Liang et al., 2016). The aforementioned researches provide guidance for how to avoid the negative effects on spin transport originated from molecular structure. As for element composition, Nguyen et al. have studied and compared the spin responses in different devices based on π-conjugated polymers made of protonated, H-, and deuterated, D-hydrogen. Because of D-hydrogen having a weaker hyperfine interaction, the results show that the device based on D-hydrogen obtained a longer spin diffusion length and a larger MR (Nguyen et al., 2010). Furthermore, the effect of the aggregation structures of OSCs on spin transport property has been researched. By controlling the substrate temperature when thermally evaporating the molecular layer, Sun et al. have fabricated two types of spin valves based on polycrystalline and amorphous fluorinated copper phthalocyanine (F_16_CuPc) thin film, respectively (Figure 1D) (Sun et al., 2016b). By comparing the magnetoresistance results of the two types of devices, distinctly, the spin valve with amorphous thin film of F_16_CuPc possesses larger MR and longer spin transport length due to the lower spin scattering from the grain boundary and greater resistance to the top electrode penetration.

In addition to spin valve, pure spin current device is another type of device to study spin transport, which has the trilayer structure of ferromagnet/OSC/nonmagnetic (Ando et al., 2013; Jiang et al., 2015; Sun et al., 2016a). A pure spin current is a flow of electron spin angular momentum carried by electrons; however, there is no net charge current that can be detected in the external circuit because the flow direction of electrons with opposite spin orientation are opposite according to spin pumping principle. The pure spin current is injected by spin pumping through a ferromagnetic resonance in the magnetic insulator (Ando et al., 2013; Watanabe et al., 2014). Different from the hopping spin transport mechanism in spin valves, the spin transport in the pure spin current device is due to an exchange interaction between polarons at the interface, which is much faster than the carrier mobility (Jiang et al., 2015). Based on pure spin current device, Watanabe et al. have demonstrated the ability of polarons to carry pure spin currents over hundreds of nanometers with millisecond spin relaxation time (Watanabe et al., 2014). Thus, keeping the study of pure spin current is meaningful, which plays a crucial role in transmitting, processing, and storing information (Watanabe et al., 2014).

In fact, although the application of OSCs in spin transport study has been developed for more than 10 years, there are still some challenges that need to be conquered. First, the effective and universal preparation method of reliable spin valve is still lacking, which has seriously limited the progress of room-temperature operable spin valve, functional spintronic devices, and the repeatability of devices. So far, several methods directed at top electrode preparation with low damage on organic materials have been proposed, such as depositing top electrodes at cryogenic temperature (Sun et al., 2016b) and top electrode transfer method (Ding et al., 2018). Second, systematical studies on the relationship between the spin transport characteristic and material composition and structure are still few. Thus, more researches are required for enriching the theoretical and experimental foundation of organic spintronics. Third, a mass of novel materials with high mobility or unique properties, such as soluble semiconductors, organic single crystal, and co-crystal materials, are urgently needed to be applied in spin transport study (Wang et al., 2016; Tsurumi et al., 2017). However, relative technical matters should be solved before this, such as the problem of spin-scattering increasement caused by solvent residue, the vacancy formed by solvent evaporation, and the demand for novel preparation method of spintronic device based on crystal materials. Therefore, novel device fabrication techniques are urgently explored in the following research, especially the construction of reliable lateral spintronic devices; however, the progress is still slow.

## Application of OSCs in Functional Spin Devices

Aside from the excellent spin transport property, some of the OSCs also possess distinct electro-optical characteristics or flexibility, which make the OSCs applicable to the construction of multifunctional spin devices.

### Spin Memory Devices

Because of the non-linear electrical property of OSC-based spin valve, the electrical memory effect can be combined with the spin-valve effect and even for achieving the electrically controllable MR. The mechanism of the electrical memory in spin valves is the formation and break of conductive filaments, which is controlled by the external voltage and the design of interface (Wang et al., 2015). The interface is required to be a critical state, so that the filaments can easily form and break by changing the external voltage. Therefore, it is easy to realize different non-volatile resistive states, which is the typical performance of spin memory devices.

Based on the commonly used π-conjugated OSC of tris(8-hydroxyquinolinato)aluminum (Alq_3_) and the wide voltage irreversible area of device, Hueso et al. have reported the first hybrid spin valve with electrically non-volatile memory functionalities simultaneously (Hueso et al., 2007). Inspired by this work, a full electrical controllable magnetoresistance with multiple tunable non-volatile states (Prezioso et al., 2011) and its capability of information processing (Prezioso et al., 2013) have been further investigated in a similar spin valve based on the same OSC. The excellent memory ability of 32 states or 5-bit storage in this Alq_3_-based spin valve lays the foundation of multi-state storage devices.

In fact, although the existing researches amply indicate the feasibility of spin-valve effect plus electrical memory, how to accurately control the critical interface for realizing both the spin injection or detection and electrical memory is still a tough work in this direction. Several factors have influence on such critical interfacial state of the filament formation and break, such as the device preparation method, the surface roughness, and morphology of the OSC. The mixture of multiple factors makes it difficult to summarize a universal rule for reaching this critical state; therefore, a large number of experiments and more efforts are needed. In addition, only a few OSCs have been applied in spin memory devices; other diverse OSCs are eagerly expected to be used to broaden and deepen the research of this device (Yuan et al., 2019; Liu et al., 2020). Furthermore, such type of spin memory device still has a big gap for future practical application; the working stability even at room temperature will be a long-term objective.

### Spin Photoresponse Devices

**Figure 2:**
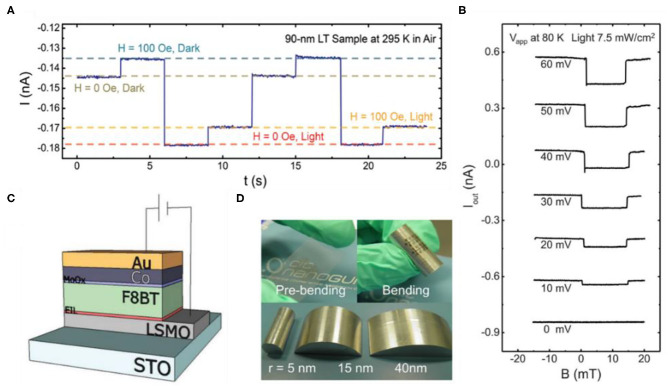
**(A)** Realization of four different resistance states through controllable switching of the magnetic field and light exposure in the same device in air (Sun et al., 2016b). **(B)** Manipulation of *I*–*B* curves under different applied biases and constant light irradiation (Sun et al., 2017). **(C)** Schematic structure of spin-OLED device (Prieto-Ruiz et al., 2019). **(D)** Photographs of the flexible semi-transparent BCP-based spin valve, the bending-tolerance test, and the curved supports employed in this work (Sun et al., 2014b).

In addition to the semiconductor characteristic, most of the OSCs also have abundant optical properties and thus can be used for sensors. Photoresponse effect is one of the common optical properties where the photogenerated carriers will influence the device resistance during the external light irradiation. On the basis of this point, F_16_CuPc, possessing photoresponse and air stability, served as the spacer of spin valve, and four resistance states can be output in a spin photoresponse device as shown in Figure 2A (Sun et al., 2016b). In this device, the photogenerated carriers are non-spin-polarized, so that the photogenerated carriers can only influence the output current but for the absolute spin-polarized current. To release the photoresponse characteristic of OSCs, several important techniques play a key role, including the application of liquid nitrogen during the fabrication of organic layer and top electrode, and the interface layer of leaky-AlO_X_, which facilitates the real spin transport occurring in F_16_CuPc and thus the photoresponse effect. Meanwhile, a large MR of about 4% and a long spin transport distance of 180 nm at room temperature have been detected, which prove the excellent properties of this device again. For the future development, more π-conjugated materials with excellent spin transport and photosensitive abilities are required to be applied in spin photoresponse devices, and novel functions may be explored concomitantly.

### Spin Photovoltaic Devices

Recently, the photovoltaic character of OSCs has also been successfully used in spintronic study. By combining the spin valve effect and photovoltaic effect together, spin photovoltaic device, one of the important multifunctional spintronic devices, has been realized. On the basis of the reliable fabrication of spintronic device and high-efficiency spin injection, the spin transport through the OSC layer should be ensured, otherwise, the spin photovoltaic effect should not be implemented.

Based on C_60_, which possesses both high electron mobility and photovoltaic property, Sun et al. have fabricated the first organic spin photovoltaic device (Sun et al., 2017). As an independent spin valve, a large MR of 6.5% has been measured at room temperature, which shows the excellent spin transport property of C_60_ and the great quality of device. In such device, the photogenerated current originated from the photovoltaic effect of C_60_ plays an important role as a modulator of output current, where the non-spin-polarized part of injected spin-polarized electrons by bias can be controllably recombined with the photogenerated holes, thus the amplitude of final output current and the MR value can be modulated (Figure 2B). The variation of the range of output current can even reach from positive to negative, and undoubtedly, the photovoltaic character of OSCs implies significant function in this device. In view of the great potential of photovoltaic property for developing spin photovoltaic device, H_2_Pc, hole-transport small molecular semiconductor, has been used as the spacer for further investigating the spin photovoltaic property (Bairagi et al., 2020). Benefiting from the stability in ambient conditions of H_2_Pc, a large MR of 7% and prominent spin photovoltaic characteristic at room temperature have been measured.

Although an increasing number of works have been reported toward spin photovoltaic device, the current situation still remains at the proof of principle and the photovoltaic effect of the used materials is too weak. At present, a number of excellent photovoltaic materials have been synthesized in the organic photovoltaic field (Yuan et al., 2019; Liu et al., 2020). However, many challenges remain in the application of these materials in spin photovoltaic device; for instance, most of these materials can only be formed as membrane via solution processing, which may easily lead to residual solvent and thus the spin scattering. Therefore, novel techniques and OSCs are still needed for improving both the photovoltaic effect and the spin transport ability in spin photovoltaic devices.

### Spin-OLED

The electroluminescent (EL) property of OSCs, which has already been applied in organic light-emitting diodes (OLED), can also be used for building multifunctional spintronic devices. With the bipolar spin injection, the electroluminescence efficiency can be modulated by the relative direction of the two ferromagnetic electrodes according to the quantum efficiency mechanism. Based on Alq_3_, a frequently used OSC with light emitting property, Nguyen et al. have proposed and investigated the first spin-OLED (Nguyen et al., 2012). According to the quantum mechanism, the singlet probability can reach 1/2 modulated by external magnetic field, which is larger than the normal OLED with non-magnetic electrodes (Bergenti et al., 2004), so that the luminous efficiency can be enhanced by spin injection. The device shows the magneto-electroluminescence (MEL) of 1% at bias voltage of 3.5 V and 10 K, with the coercive fields of electrodes adjusted by the emission intensity. The key of the achievement is that they choose a deuterated organic polymer interlayer of poly(phenylene-vinylene) (H-DOO-PPV), which has a weak hyperfine interaction and hence excellent spin transport property (Nguyen et al., 2010). The spin diffusion length of deuterated poly(dioctyloxy)phenyl vinylene (D-DOO-PPV) is about 45 nm, which is about three times more than that of H-DOO-PPV polymer (Nguyen et al., 2012). Also, they inserted a thin buffer layer of LiF to improve the efficiency of injection (Schulz et al., 2011). Afterwards, for enhancing the MEL, Prieto-Ruiz et al. selected the light-emitting conjugated polymer poly(9,9-dioctylfluorene-co-benzothidiazole) (F8BT) as the spacer because of its high green EL intensity and bipolar character (Figure 2C) (Prieto-Ruiz et al., 2019). The device realizes spin valve effect in spin-OLED at high bias voltage of 14 V through a careful engineering of the organic/inorganic interfaces, so that the MEL is enhanced on the order of 2.4% at 9 V and 20 K, based on the antiparallel configuration of the ferromagnetic electrodes.

These works offer more possibilities of multifunctional spintronic devices. However, as a spin-OLED, higher MEL are required to break through by optimizing the interface and enhancing the efficiency of spin injection. Moreover, operation at room temperature is still a goal in this area.

### Flexible Spin Devices

OSCs with mechanical flexibility are widely used in preparing flexible organic electronic devices. However, there are a few studies on flexible spintronic devices. The first flexible spin valve was fabricated based on bathocuproine (BCP), and a large MR of 3.5% is observed at room temperature (Sun et al., 2014b). More importantly, the spin valve has shown excellent stability after the endurance measurements of the bending radius and the bending time (Figure 2D). Both the *I*–*V* and the MR characteristics are almost unchanged, even up to a bending radius of 5 mm or after bending for 50 times.

The aforementioned work has shown a beginning toward application; however, the thermal-evaporated OSCs apparently limit the actual use, while solution-processed OSCs are the mainstream in the flexible application. Moreover, the requirement of room-temperature stable operation has also posed a challenge in this type of spintronic device.

## Organic-Based Magnets as the Magnetic Electrodes

The conductivity mismatch between ferromagnetic electrodes and OSC thin films is a main factor for limiting the spin injection efficiency (Schmidt et al., 2000). Utilizing organic-based magnets to replace ferromagnetic electrode, which has lower energy level, is an efficient method to avoid this problem (Yoo et al., 2010).

**Figure 3:**
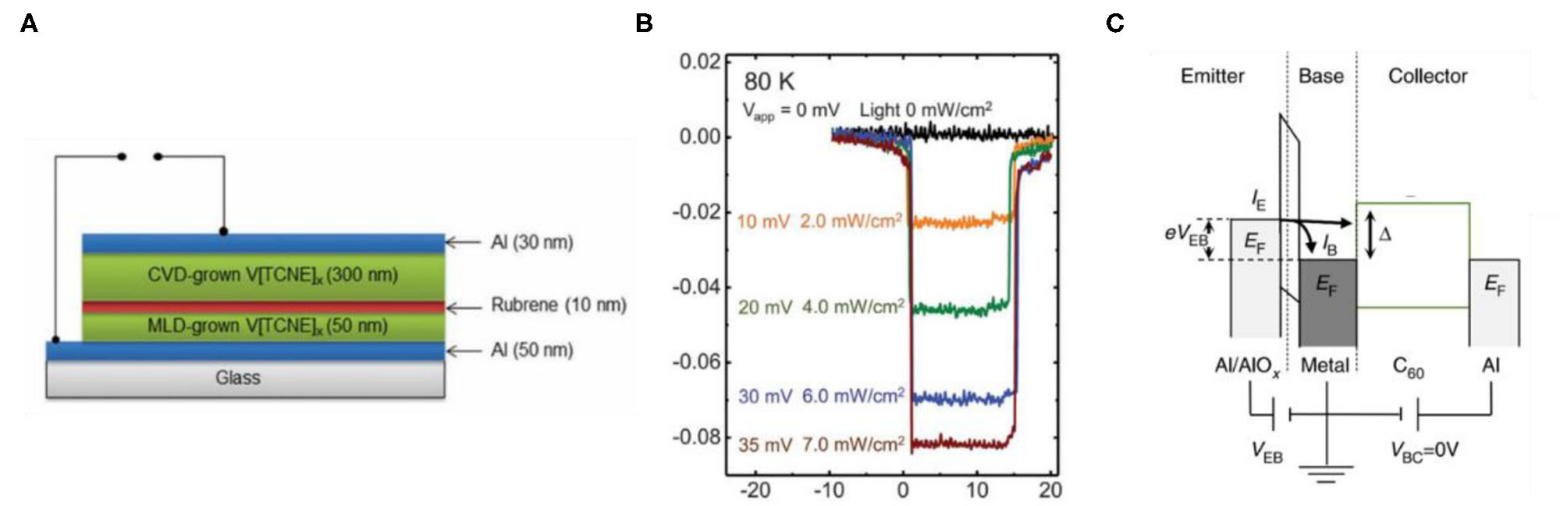
**(A)** Device structure of Al/V[TCNE]_x_/rubrene/V[TCNE]_x_/Al (Li et al., 2011). **(B)** Electro-optical modulation, varying both the applied voltage bias and the light irradiation (Sun et al., 2017). **(C)** The energy diagram and structure of a C_60_-based hot-electron spin transistor (Gobbi et al., 2014).

V[TCNE]_x_ (*x* ≈ 2, TCNE: tetracyanoethylene), which possesses the merits of high magnetic ordering temperature, fully spin-polarized semiconducting electronic structure, chemical tunability, and low-temperature processing, was first applied as one of the electrodes to fabricate spin valves (Manriquez et al., 1991; Yoo et al., 2010). Based on the MR measurements of temperature dependence and bias dependence, the results suggest that the organic-based magnets can be used as a spin injector or detector. After this, an all-organic-based spin valve has been fabricated based on the same organic magnet. The device structure is shown in Figure 3A, with two V[TCNE]_x_ layers as the injector and detector, respectively (Li et al., 2011). A new model of spin-dependent tunneling between highly spin-polarized band can successfully demonstrate the process of spin injection and detection in the all-organic spin valves. Recently, an all-organic dual spin valve with three organic spin-selective layers has been reported, which introduced single molecular magnets of manganese and cobalt phthalocyanines (MnPc and CoPc) as the injector and detector (Banerjee and Pal, 2018). There are four configurations with two spin-flip interfaces at most. When the injector and detector have asymmetric thickness and different single molecular magnets, it can achieve four separate resistive states as 2-bit logic.

Although the previously mentioned applied organic-based magnets have shown promising application in spintronic study, more novel organic-based magnets are still needed to be developed and used in this direction. Moreover, organic-based magnets also have a potential in the application of flexible devices, and the endurance measurement and bending strength measurement are required to be filled up in the future study.

## Spin Manipulation in OSCs

It is of great significance to achieve spin manipulation in OSCs, and strong SOC and Hanle effect are the basis of spin manipulation in a traditional way. The weak SOC interaction of OSCs, on the one hand, contributes to the long-distance spin transport, but on the other hand, it makes spin manipulation particularly difficult. So far, the output spin signal can be preliminarily controlled based on spin photovoltaic device reported by Sun et al. (2017) (Figure 3B), which indicates that the multifunctional spintronic device depending on the photoelectric properties of OSCs has presented a possibility of spin manipulation. However, it is just a simple and primary idea, which still needs further detailed research.

In addition, starting from the device structure design, the hot-electron spin transistor with three-terminal structure has also shown a promising application in spin manipulation (Figure 3C) (Appelbaum et al., 2007; Huang et al., 2007; Appelbaum, 2011; Gobbi et al., 2014). Through two spin injection ways with opposite direction, hot-electron spin injection and electric spin injection, a controllable spin-polarized current may be finally output. However, for ensuring both efficient spin injection in two ways, the preparation level of such device needs to be improved greatly.

## Conclusion

In conclusion, the application of OSCs in the aspects of spin transport, spin functional devices, all-organic spin devices, and spin manipulation has been introduced in this review (Table 1). Also, the advanced achievements and obstacles for further development are discussed. For the future, according to the other achievements in organic electronic fields and spintronics, more types of OSCs are required to be applied, such as the interfacial materials, high-mobility materials, organic single crystals, supramolecules, and thermoelectric materials. Furthermore, taking advantage of the OSCs, novel multifunctional spintronic devices need to be urgently explored, for instance, the combination of organic spin valve and field effect transistor, which are always pursued in organic spintronics. With the development of organic spintronics, more strategies of OSC application will be explored to overcome the present challenges and keep this field attractive and active.

**Table 1:** A summarized table of organic semiconductor applications in spintronics with the corresponding references.

**Application**	**Device structure (bottom to top)**	**Temperature (K)**	**References**
Spin valve	LSMO/**PIID-CNTVT-C1**/Ni_80_Fe_20_/Au LSMO/**PIID-CNTVT-C3**/Ni_80_Fe_20_/Au LSMO/**PAIID-CNTVT-C1**/Ni_80_Fe_20_/Au LSMO/**PAIID-CNTVT-C3**/Ni_80_Fe_20_/Au	50–200	Li et al., 2019
	LSMO/**C**_**60**_/Co/Al LSMO/**C**_**70**_/Co/Al	20–300	Liang et al., 2016
	LSMO/**DOO-PPV**/Co	10–300	Nguyen et al., 2010
	Co/AlO_x_/**F**_**16**_**CuPc**/Ni_80_Fe_20_	7–295	Sun et al., 2016b
Pure spin current devices	Ni_80_Fe_20_/**PBTTT**/Pt	200–300	Watanabe et al., 2014
Spin memory	LSMO/**Alq**_**3**_/AlO_x_/Co	100–300	Prezioso et al., 2011
Spin photoresponse	Co/AlO_x_/**F**_**16**_**CuPc**/Ni_80_Fe_20_	7–295	Sun et al., 2016b
Spin photovoltaic	Co/AlO_x_/**C**_**60**_/Ni_80_Fe_20_	80–295	Sun et al., 2017
	Co/AlO_x_/**H**_**2**_**Pc**/Ni_80_Fe_20_	300	Bairagi et al., 2020
Spin-OLED	LSMO/**DOO-PPV**/LiF/Co/Al	10–300	Nguyen et al., 2012
	LSMO/PEIE/**F8BT**/MoO_x_/Co/Au	20–200	Prieto-Ruiz et al., 2019
Flexible spin devices	Co/AlO_x_/**BCP**/Ni_80_Fe_20_	300	Sun et al., 2014b
All-organic spin devices	Al/**V[TCNE]**_**x**_/**rubrene**/**V[TCNE]**_**x**_/Al	120–200	Li et al., 2011
Spin manipulation	Co/AlO_x_/**C**_**60**_/Ni_80_Fe_20_	80–295	Sun et al., 2017

*Organic semiconductors are in bold type*.

## Author Contributions

YZ wrote the paper. LG and XZ completed the spelling and grammar check and copyright section. LG and XS supervised this review and completed all the submissions. All authors joined the discussion and revision of this paper.

## Conflict of Interest

The authors declare that the research was conducted in the absence of any commercial or financial relationships that could be construed as a potential conflict of interest.

## References

[B1] Ando K. Watanabe S. Mooser S. Saitoh E. Sirringhaus H. (2013). Solution-processed organic spin-charge converter. Nat. Mater. 12, 622–627. 10.1038/nmat3634 23644525

[B2] Appelbaum I. (2011). Introduction to spin-polarized ballistic hot electron injection and detection in silicon. Philos. Trans. R. Soc. A 369, 3554–3574. 10.1098/rsta.2011.0137 21859721

[B3] Appelbaum I. Huang B. Monsma D. J. (2007). Electronic measurement and control of spin transport in silicon. Nature 447, 295–298. 10.1038/nature05803 17507978

[B4] Baibich M. N. Broto J. M. Fert A. Nguyen Van Dau F. Petroff F. Etienne P.. (1988). Giant magnetoresistance of (001)Fe/(001)Cr magnetic superlattices. Phys. Rev. Lett. 61, 2472–2475. 10.1103/PhysRevLett.61.2472 10039127

[B5] Bairagi K. Romero D. G. Calavalle F. Catalano S. Zuccatti E. Llopis R.. (2020). Room-temperature operation of a p-type molecular spin photovoltaic device on a transparent substrate. Adv. Mater. 32:e1906908. 10.1002/adma.201906908 31944432

[B6] Banerjee A. Pal A. J. (2018). All-organic dual spin valves with well-resolved four resistive-states. Small 14:e1801510. 10.1002/smll.201801510 29998514

[B7] Bergenti I. Dediu V. Arisi E. Mertelj T. Murgia M. Riminucci A. (2004). Spin polarised electrodes for organic light emitting diodes. Org. Electron. 5, 309–314. 10.1016/j.orgel.2004.10.004

[B8] Boehme C. Lupton J. M. (2013). Challenges for organic spintronics. Nat. Nanotechnol. 8, 612–615. 10.1038/nnano.2013.177 24002071

[B9] Dediu V. A. Hueso L. E. Bergenti I. Taliani C. (2009). Spin routes in organic semiconductors. Nat. Mater. 8, 707–716. 10.1038/nmat2510 19701216

[B10] Ding S. Tian Y. Wang H. Zhou Z. Mi W. Ni Z.. (2018). Reliable spin valves of conjugated polymer based on mechanically transferrable top electrodes. ACS Nano 12, 12657–12664. 10.1021/acsnano.8b07468 30412379

[B11] Gobbi M. Pietrobon L. Atxabal A. Bedoya-Pinto A. Sun X. Golmar F.. (2014). Determination of energy level alignment at metal/molecule interfaces by in-device electrical spectroscopy. Nat. Commun. 5:4161. 10.1038/ncomms5161 24946715

[B12] Guo L. Gu X. Zhu X. Sun X. (2019a). Recent advances in molecular spintronics: multifunctional spintronic devices. Adv. Mater. 31:e1805355. 10.1002/adma.201805355 30680807

[B13] Guo L. Qin Y. Gu X. Zhu X. Zhou Q. Sun X. (2019b). Spin transport in organic molecules. Front. Chem. 7:428. 10.3389/fchem.2019.00428 31275920 PMC6591472

[B14] Huang B. Monsma D. J. Appelbaum I. (2007). Coherent spin transport through a 350 micron thick silicon wafer. Phys. Rev. Lett. 99:177209. 10.1103/PhysRevLett.99.177209 17995369

[B15] Hueso L. E. Bergenti I. Riminucci A. Zhan Y. Q. Dediu V. (2007). Multipurpose magnetic organic hybrid devices. Adv. Mater. 19, 2639–2642. 10.1002/adma.200602748

[B16] Jang H. J. Richter C. A. (2017). Organic spin-valves and beyond: spin injection and transport in organic semiconductors and the effect of interfacial engineering. Adv. Mater. 29, 1602739. 10.1002/adma.201602739 27859663

[B17] Jiang S. W. Liu S. Wang P. Luan Z. Z. Tao X. D. Ding H. F.. (2015). Exchange-dominated pure spin current transport inAlq3Molecules. Phys. Rev. Lett. 115:086601. 10.1103/PhysRevLett.115.086601 26340196

[B18] Li B. Kao C. Y. Yoo J. W. Prigodin V. N. Epstein A. J. (2011). Magnetoresistance in an all-organic-based spin valve. Adv. Mater. 23, 3382–3386. 10.1002/adma.201100903 21721052

[B19] Li D. Wang X. Lin Z. Zheng Y. Jiang Q. Zheng N.. (2019). Tuning charge carrier and spin transport properties via structural modification of polymer semiconductors. ACS Appl. Mater. Inter. 11, 30089–30097. 10.1021/acsami.9b07863 31342737

[B20] Li F. Li T. Chen F. Zhang F. (2015). Excellent spin transport in spin valves based on the conjugated polymer with high carrier mobility. Sci. Rep. 5:9355. 10.1038/srep09355 25797862 PMC4369752

[B21] Liang S. Geng R. Yang B. Zhao W. Chandra Subedi R. Li X.. (2016). Curvature-enhanced spin-orbit coupling and spinterface effect in fullerene-based spin valves. Sci. Rep. 6:19461. 10.1038/srep19461 26786047 PMC4726316

[B22] Liu Q. Jiang Y. Jin K. Qin J. Xu J. Li W. (2020). 18% Efficiency organic solar cells. Sci. Bull. 65, 272–275. 10.1016/j.scib.2020.01.001 36659090

[B23] Manriquez J. M. Yee G. T. McLean R. S. Epstein A. J. Miller J. S. (1991). A room-temperature molecular/organic-based magnet. Science 252, 1415–1417. 10.1126/science.252.5011.1415 17772914

[B24] Nguyen T. D. Ehrenfreund E. Vardeny Z. V. (2012). Spin-polarized light-emitting diode based on an organic bipolar spin valve. Science 337, 204–209. 10.1126/science.1223444 22798608

[B25] Nguyen T. D. Hukic-Markosian G. Wang F. Wojcik L. Li X. G. Ehrenfreund E.. (2010). Isotope effect in spin response of pi-conjugated polymer films and devices. Nat. Mater. 9, 345–352. 10.1038/nmat2633 20154693

[B26] Prezioso M. Riminucci A. Bergenti I. Graziosi P. Brunel D. Dediu V. A. (2011). Electrically programmable magnetoresistance in multifunctional organic-based spin valve devices. Adv. Mater. 23, 1371–1375. 10.1002/adma.201003974 21400598

[B27] Prezioso M. Riminucci A. Graziosi P. Bergenti I. Rakshit R. Cecchini R.. (2013). A single-device universal logic gate based on a magnetically enhanced memristor. Adv. Mater. 25, 534–538. 10.1002/adma.201202031 23097157

[B28] Prieto-Ruiz J. P. Miralles S. G. Prima-García H. López-Muñoz A. Riminucci A. Graziosi P.. (2019). Enhancing light emission in interface engineered spin-OLEDs through spin-polarized injection at high voltages. Adv. Mater. 31:1806817. 10.1002/adma.201806817 30645012

[B29] Schmidt G. Ferrand D. Molenkamp L. W. (2000). Fundamental obstacle for electrical spin injection from a ferromagnetic metal into a diffusive semiconductor. Phys. Rev. B 62, 4790–4793. 10.1103/PhysRevB.62.R4790

[B30] Schulz L. Nuccio L. Willis M. Desai P. Shakya P. Kreouzis T.. (2011). Engineering spin propagation across a hybrid organic/inorganic interface using a polar layer. Nat. Mater. 10, 39–44. 10.1038/nmat2912 21131962

[B31] Sun D. Ehrenfreund E. Vardeny Z. V. (2014a). The first decade of organic spintronics research. Chem. Commun. 50, 1781–1793. 10.1039/C3CC47126H 24432354

[B32] Sun D. van Schooten K. J. Kavand M. Malissa H. Zhang C. Groesbeck M.. (2016a). Inverse spin hall effect from pulsed spin current in organic semiconductors with tunable spin-orbit coupling. Nat. Mater. 15, 863–869. 10.1038/nmat4618 27088233

[B33] Sun X. Bedoya-Pinto A. Llopis R. Casanova F. Hueso L. E. (2014b). Flexible semi-transparent organic spin valve based on bathocuproine. Appl. Phys. Lett. 105:083302 10.1063/1.4894114

[B34] Sun X. Bedoya-Pinto A. Mao Z. Gobbi M. Yan W. Guo Y.. (2016b). Active morphology control for concomitant long distance spin transport and photoresponse in a single organic device. Adv. Mater. 28, 2609–2615. 10.1002/adma.201503831 26823157

[B35] Sun X. Gobbi M. Bedoya-Pinto A. Txoperena O. Golmar F. Llopis R. (2013). Room-temperature air-stable spin transport in bathocuproine-based spin valves. Nat. Commun. 4:2794 10.1038/ncomms3794

[B36] Sun X. Velez S. Atxabal A. Bedoya-Pinto A. Parui S. Zhu X.. (2017). A molecular spin-photovoltaic device. Science 357, 677–680. 10.1126/science.aan5348 28818941

[B37] Tsurumi J. Matsui H. Kubo T. Häusermann R. Mitsui C. Okamoto T. (2017). Coexistence of ultra-long spin relaxation time and coherent charge transport in organic single-crystal semiconductors. Nat. Phys. 13, 994–998. 10.1038/nphys4217

[B38] Wang L. Yang C. Wen J. Gai S. Peng Y. (2015). Overview of emerging memristor families from resistive memristor to spintronic memristor. J. Mater. Sci. Mater. Electron. 26, 4618–4628. 10.1007/s10854-015-2848-z

[B39] Wang Y. Zhu W. Dong H. Zhang X. Li R. Hu W. (2016). Organic cocrystals: new strategy for molecular collaborative innovation. Top Curr. Chem. 374:83. 10.1007/s41061-016-0081-8 27885589

[B40] Watanabe S. Ando K. Kang K. Mooser S. Vaynzof Y. Kurebayashi H. (2014). Polaron spin current transport in organic semiconductors. Nat. Phys. 10, 308–313. 10.1038/nphys2901

[B41] Wolf S. A. Awschalom D. D. Buhrman R. A. Daughton J. M. von Molnar S. Roukes M. L.. (2001). Spintronics: a spin-based electronics vision for the future. Science 294, 1488–1495. 10.1126/science.1065389 11711666

[B42] Yoo J. W. Chen C. Y. Jang H. W. Bark C. W. Prigodin V. N. Eom C. B.. (2010). Spin injection/detection using an organic-based magnetic semiconductor. Nat. Mater. 9, 638–642. 10.1038/nmat2797 20639895

[B43] Yuan J. Zhang Y. Zhou L. Zhang G. Yip H.-L. Lau T.-K. (2019). Single-junction organic solar cell with over 15% efficiency using fused-ring acceptor with electron-deficient core. Joule 3, 1140–1151. 10.1016/j.joule.2019.01.004

[B44] Zhang X. Mizukami S. Kubota T. Ma Q. Oogane M. Naganuma H.. (2013). Observation of a large spin-dependent transport length in organic spin valves at room temperature. Nat. Commun. 4:1392. 10.1038/ncomms2423 23340432

